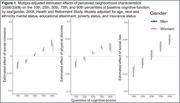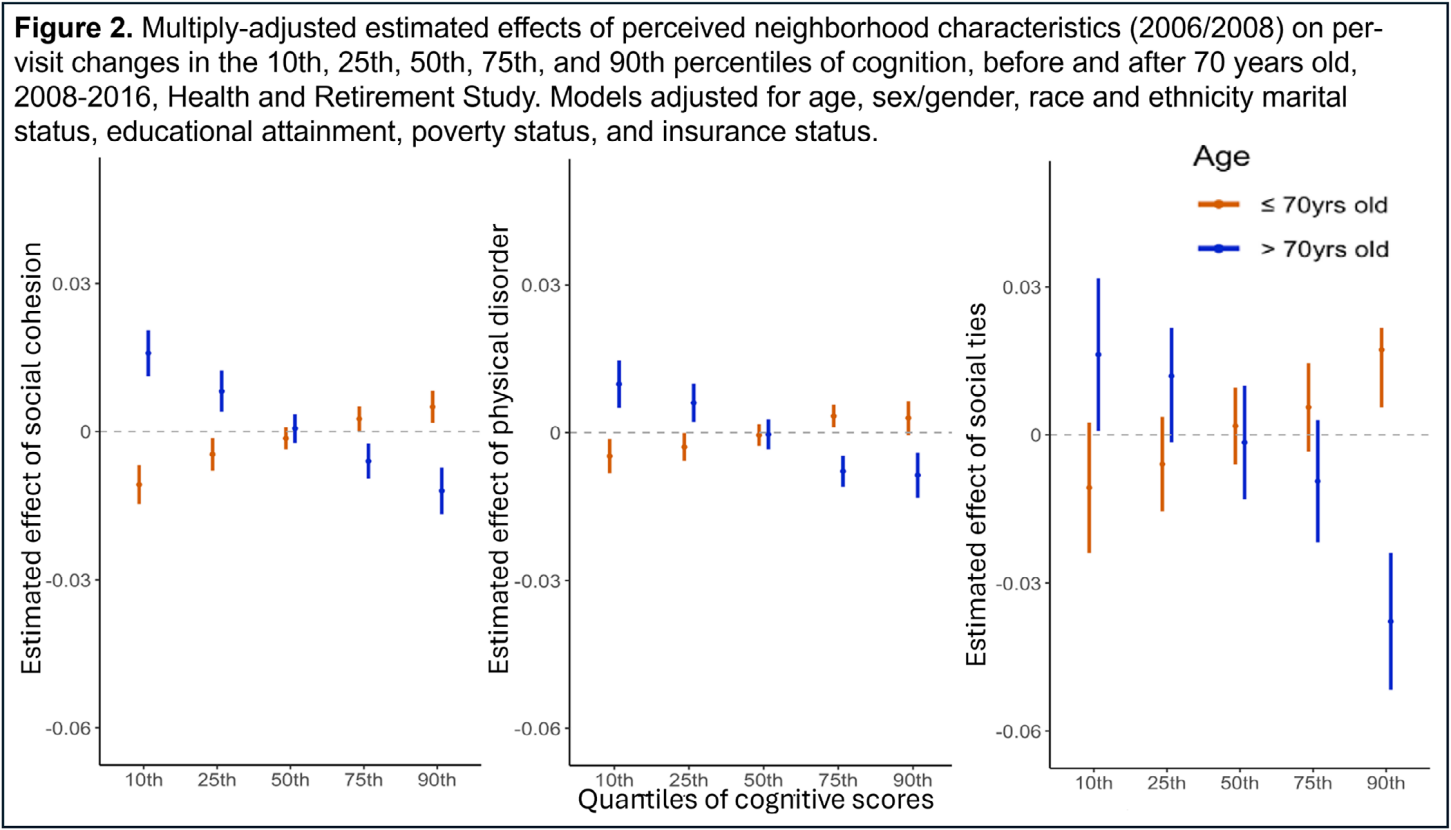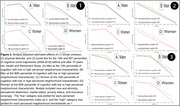# Perceived neighborhood characteristics and cognition function in the Health and Retirement Study: a quantile regression analysis

**DOI:** 10.1002/alz70860_101174

**Published:** 2025-12-23

**Authors:** Kendra D Sims

**Affiliations:** ^1^ Boston University, Boston, MA, USA

## Abstract

**Background:**

By facilitating health‐promoting behaviors and reducing cardiovascular risk, positive perceptions about neighborhood conditions have been associated with slower cognitive decline. We aimed to evaluate the impact of these psychosocial resources across the continuum of cognition. We hypothesized that older women at elevated dementia risk (i.e., lower quantiles of cognition) receive more cognitive benefit than men from perceiving safer, cohesive neighborhoods and more interpersonal ties.

**Method:**

We used Health and Retirement Study participants (*n* = 12,341, aged 52‐104, 58.7% women, 78.0% Non‐Hispanic White) with data on our biennially‐collected outcome of interest: averaged 10‐word immediate and delayed word recall scores. Our three primary exposures were reported social cohesion, physical disorder, and social ties within the immediate area in 2006 or 2008. We estimated linear quantile models as well as linear quantile mixed effects models for associations of each perceived neighborhood condition with level (2008) along with change (2008‐2016) in word recall across 10th, 25th, 50th, 75th and 90th percentiles. We evaluated heterogeneity by sex/gender and age (≤ versus > 70 years).

**Result:**

Men and women scored comparably on baseline word recall (median 5 (IQR: 4, 6)). Perceiving positive neighborhood conditions was associated with better baseline cognitive function at quantiles indicating lowest risk for dementia (e.g., β_90th_ for lower physical disorder = 0.108, 95% CI = 0.052 to 0.164)). At cognitive quantiles indicative of worse function, higher neighborhood cohesion (β_10th_ = 0.016, 95% CI = 0.011 to 0.021), more social ties (β_10th_ = 0.016, 95% CI = 0.001 to 0.032) and lower neighborhood disorder (β_10th_ = 0.010, 95% CI = 0.005 to 0.015) were each associated with slower per‐visit decline after 70. After age 70, associations of more versus fewer social ties with steeper annual cognitive decline were evident among women at lower dementia risk (β_75th_: ‐0.062 versus β_75th_: ‐0.054, p for trend: 0.022) but not men (β_75th_:‐0.052 versus β_75th_:‐0.053, p for trend: 0.724).

**Conclusion:**

Positive perceptions of the neighborhood environment may slow decline for older adults at risk of dementia. Future work should contextualize associations among women by evaluating gender‐specific social ties along with intermediate mechanisms between neighborhood context and dementia risk.